# 1-(3,5-Di­fluoro­phen­yl)-4,4,4-tri­fluoro­butane-1,3-dione

**DOI:** 10.1107/S1600536813028535

**Published:** 2013-10-26

**Authors:** K.E. Manoj Kumar, B. S. Palakshamurthy, P. A. Suchetan, S. Madan Kumar, N.K. Lokanath, S. Sreenivasa

**Affiliations:** a Department of Studies and Research in Chemistry, Tumkur University, Tumkur, Karnataka 572 103, India; b Department of Studies and Research in Physics, U.C.S., Tumkur University, Tumkur, Karnataka 572 103, India; c Department of Studies and Research in Chemistry, U.C.S., Tumkur University, Tumkur, Karnataka 572 103, India; d Department of Studies in Physics, University of Mysore, Manasagangotri, Mysore, India

## Abstract

In the title compound, C_10_H_5_F_5_O_2_, the C=O bonds are *syn* to one another. In the crystal, mol­ecules are linked into *C*(9) chains parallel to [101] through weak C—H⋯O inter­actions, with the O atom adjacent to the –CF_3_ group acting as the acceptor.

## Related literature

For biological-activity studies of compounds with tri­fluoro­methyl substituents, see: Manoj Kumar *et al.* (2013 ▶).
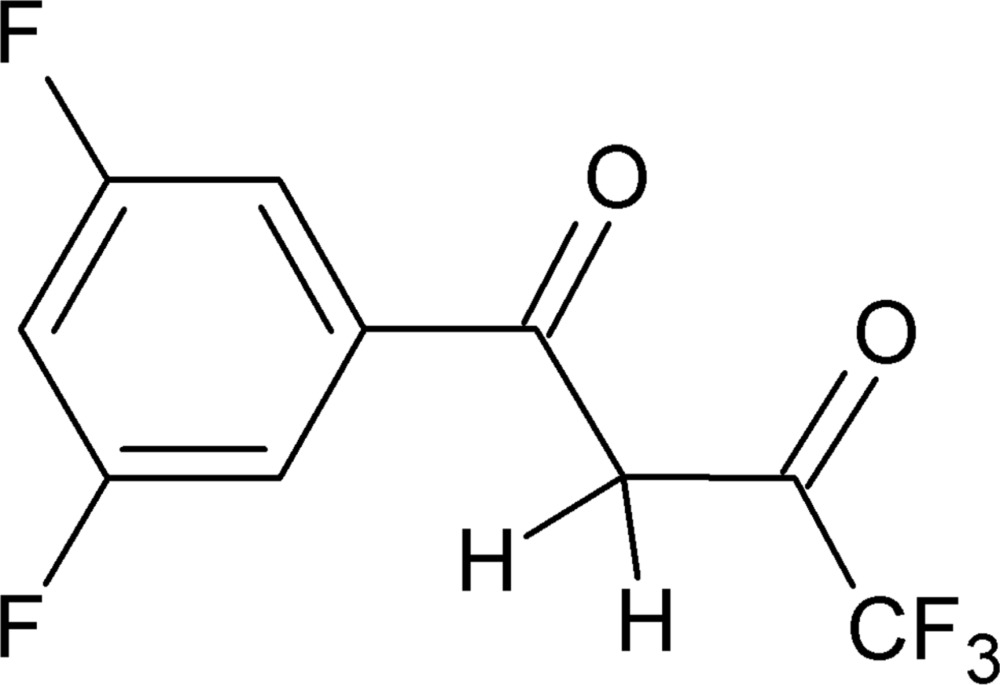


## Experimental

### 

#### Crystal data

C_10_H_5_F_5_O_2_
*M* *_r_* = 252.14
Monoclinic, 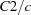

*a* = 12.393 (4) Å
*b* = 13.433 (5) Å
*c* = 12.877 (5) Å
β = 112.49 (2)°
*V* = 1980.7 (13) Å^3^
*Z* = 8
Mo *K*α radiation
μ = 0.18 mm^−1^
*T* = 294 K
0.24 × 0.20 × 0.16 mm

#### Data collection

Bruker APEXII CCD diffractometer
Absorption correction: multi-scan (*SADABS*; Bruker, 2009 ▶) *T*_min_ = 0.959, *T*_max_ = 0.972
5651 measured reflections
1604 independent reflections
1246 reflections with *I* > 2σ(*I*)
*R*_int_ = 0.040

#### Refinement

*R*[*F* ^2^ > 2σ(*F* ^2^)] = 0.074
*wR*(*F* ^2^) = 0.239
*S* = 1.08
1604 reflections
154 parameters
H-atom parameters constrained
Δρ_max_ = 0.43 e Å^−3^
Δρ_min_ = −0.47 e Å^−3^

### 

Data collection: *APEX2* (Bruker, 2009 ▶); cell refinement: *SAINT-Plus* (Bruker, 2009 ▶); data reduction: *SAINT-Plus*; program(s) used to solve structure: *SHELXS97* (Sheldrick, 2008 ▶); program(s) used to refine structure: *SHELXL97* (Sheldrick, 2008 ▶); molecular graphics: *Mercury* (Macrae *et al.*, 2008 ▶); software used to prepare material for publication: *SHELXL97*.

## Supplementary Material

Crystal structure: contains datablock(s) I, New_Global_Publ_Block. DOI: 10.1107/S1600536813028535/hb7151sup1.cif

Structure factors: contains datablock(s) I. DOI: 10.1107/S1600536813028535/hb7151Isup2.hkl

Supplementary material file. DOI: 10.1107/S1600536813028535/hb7151Isup3.cml

Additional supplementary materials:  crystallographic information; 3D view; checkCIF report

## Figures and Tables

**Table 1:** Hydrogen-bond geometry (Å, °)

*D*—H⋯*A*	*D*—H	H⋯*A*	*D*⋯*A*	*D*—H⋯*A*
C3—H3⋯O2^i^	0.93	2.53	3.462 (5)	177

Symmetry code: (i) 
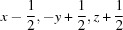
.

**Fig. 1.**
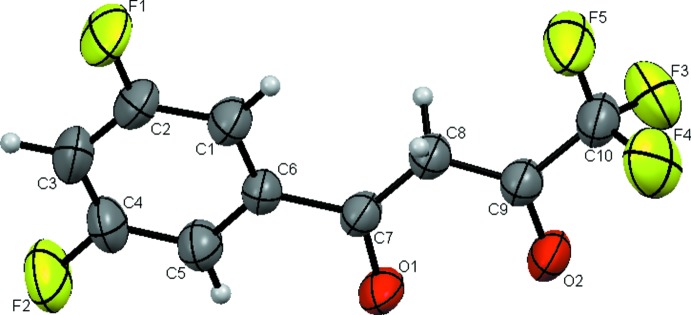
Molecular structure of the title compound, showing displacement ellipsoids drawn at the 50% probability level.

**Fig. 2.**
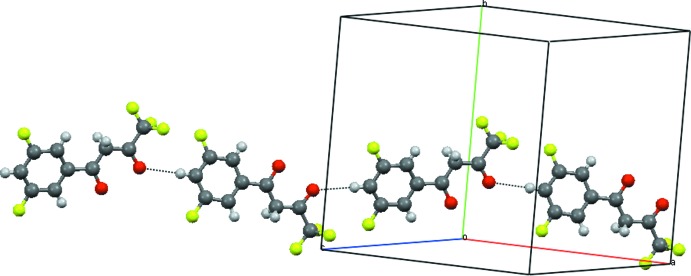
Molecular packing foming C(9) chains parallel to [101] with hydrogen bonding shown as dashed lines.

## supplementary crystallographic information

### 1. Comment

As part of our ongoing studies of the biological activities of compounds with a trifluoromethyl substituent (Manoj Kumar *et al.*, 2013), we now describe the structure of the title compound, (I).

The two C=O bonds are *syn* to one another (Fig.1), the O1—C7—C8—C9 and O2—C9—C8—C7 torsion angles being 0.4 (5) and -0.3 (6)°, respectively. In the crystal, the molecules are linked into C(9) chains parallel to [101] through a weak C3—H3···O2 interaction (Fig.2).

### 2. Experimental

3,5-Difluoroacetophenone (1 mmol) and sodium hydride (1.5 mmol) were taken in dry THF (20 ml), and the solution was stirred for 30 min at 0°C. To this solution trifluoroethylacetate (1.2 mmol) was added and the reaction mixture was stirred for 10 h at room temperature under nitrogen atmosphere. The reaction was monitored by TLC. The crude mass was purified by column chromatography using petroleum ether and ethyl acetate as an eluent (7:3), to obtain a yellow solid. Pale yellow prisms were obtained by recrystallisation fron dichloromethane/methanol (9:1) solution.

### 3. Refinement

The H atoms were positioned with idealized geometry using a riding model with C—H = 0.93–0.97 Å. All H atoms were refined with isotropic displacement parameters (set to 1.2 times of the *U*_eq_ of the parent atom).

### Crystal data

**Table:**

C_10_H_5_F_5_O_2_	Prism
*M**_r_* = 252.14	*D*_x_ = 1.691 Mg m^−^^3^
Monoclinic, *C*2/*c*	Melting point: 393 K
Hall symbol: -C 2yc	Mo *K*α radiation, λ = 0.71073 Å
*a* = 12.393 (4) Å	Cell parameters from 1023 reflections
*b* = 13.433 (5) Å	θ = 0.0–24.6°
*c* = 12.877 (5) Å	µ = 0.18 mm^−^^1^
β = 112.49 (2)°	*T* = 294 K
*V* = 1980.7 (13) Å^3^	Prism, colourless
*Z* = 8	0.24 × 0.20 × 0.16 mm
*F*(000) = 1008	

### Data collection

**Table:**

Bruker APEXII CCD diffractometer	1604 independent reflections
Radiation source: fine-focus sealed tube	1246 reflections with *I* > 2σ(*I*)
Graphite monochromator	*R*_int_ = 0.040
phi and ω scans	θ_max_ = 24.6°, θ_min_ = 2.3°
Absorption correction: multi-scan (*SADABS*; Bruker, 2009)	*h* = −14→14
*T*_min_ = 0.959, *T*_max_ = 0.972	*k* = −15→7
5651 measured reflections	*l* = −14→14

### Refinement

**Table:**

Refinement on *F*^2^	Primary atom site location: structure-invariant direct methods
Least-squares matrix: full	Secondary atom site location: difference Fourier map
*R*[*F*^2^ > 2σ(*F*^2^)] = 0.074	Hydrogen site location: inferred from neighbouring sites
*wR*(*F*^2^) = 0.239	H-atom parameters constrained
*S* = 1.08	*w* = 1/[σ^2^(*F*_o_^2^) + (0.1433*P*)^2^ + 2.2069*P*] where *P* = (*F*_o_^2^ + 2*F*_c_^2^)/3
1604 reflections	(Δ/σ)_max_ = 0.012
154 parameters	Δρ_max_ = 0.43 e Å^−^^3^
0 restraints	Δρ_min_ = −0.47 e Å^−^^3^

### Special details

**Table:**

Geometry. All e.s.d.'s (except the e.s.d. in the dihedral angle between two l.s. planes) are estimated using the full covariance matrix. The cell e.s.d.'s are taken into account individually in the estimation of e.s.d.'s in distances, angles and torsion angles; correlations between e.s.d.'s in cell parameters are only used when they are defined by crystal symmetry. An approximate (isotropic) treatment of cell e.s.d.'s is used for estimating e.s.d.'s involving l.s. planes.
Refinement. Refinement of *F*^2^ against ALL reflections. The weighted *R*-factor *wR* and goodness of fit *S* are based on *F*^2^, conventional *R*-factors *R* are based on *F*, with *F* set to zero for negative *F*^2^. The threshold expression of *F*^2^ > σ(*F*^2^) is used only for calculating *R*-factors(gt) *etc*. and is not relevant to the choice of reflections for refinement. *R*-factors based on *F*^2^ are statistically about twice as large as those based on *F*, and *R*- factors based on ALL data will be even larger.

### Fractional atomic coordinates and isotropic or equivalent isotropic displacement parameters (Å^2^)

**Table:**

	*x*	*y*	*z*	*U*_iso_*/*U*_eq_	
C1	0.0373 (3)	0.3441 (3)	0.4970 (3)	0.0558 (9)	
H1	0.0691	0.4058	0.4923	0.067*	
C2	−0.0308 (3)	0.3323 (3)	0.5594 (3)	0.0624 (10)	
C3	−0.0787 (4)	0.2426 (3)	0.5705 (3)	0.0684 (11)	
H3	−0.1238	0.2360	0.6135	0.082*	
C4	−0.0565 (4)	0.1636 (3)	0.5147 (4)	0.0649 (10)	
C5	0.0106 (3)	0.1700 (3)	0.4506 (3)	0.0574 (9)	
H5	0.0242	0.1143	0.4144	0.069*	
C6	0.0574 (3)	0.2624 (2)	0.4416 (3)	0.0474 (8)	
C7	0.1298 (3)	0.2699 (2)	0.3734 (3)	0.0476 (8)	
C8	0.1640 (3)	0.3603 (3)	0.3419 (3)	0.0511 (8)	
H8A	0.0937	0.3983	0.3019	0.061*	
H8B	0.2077	0.3967	0.4101	0.061*	
C9	0.2304 (3)	0.3596 (3)	0.2760 (3)	0.0515 (9)	
C10	0.2656 (3)	0.4562 (3)	0.2375 (3)	0.0624 (10)	
F1	−0.0500 (3)	0.4121 (2)	0.6129 (3)	0.1000 (11)	
F2	−0.1027 (3)	0.0740 (2)	0.5224 (3)	0.1010 (11)	
F3	0.2245 (3)	0.4627 (2)	0.1272 (2)	0.0913 (9)	
F4	0.3807 (2)	0.4629 (2)	0.2695 (3)	0.0989 (10)	
F5	0.2306 (3)	0.5370 (2)	0.2748 (3)	0.1110 (13)	
O1	0.1598 (2)	0.18650 (19)	0.3425 (2)	0.0671 (8)	
O2	0.2645 (2)	0.2827 (2)	0.2411 (2)	0.0666 (8)	

### Atomic displacement parameters (Å^2^)

**Table:**

	*U*^11^	*U*^22^	*U*^33^	*U*^12^	*U*^13^	*U*^23^
C1	0.065 (2)	0.051 (2)	0.066 (2)	0.0018 (15)	0.0410 (17)	−0.0022 (16)
C2	0.072 (2)	0.063 (2)	0.070 (2)	0.0110 (18)	0.0473 (19)	−0.0002 (17)
C3	0.070 (2)	0.083 (3)	0.073 (2)	0.007 (2)	0.050 (2)	0.012 (2)
C4	0.072 (2)	0.059 (2)	0.081 (2)	−0.0095 (18)	0.048 (2)	0.0108 (19)
C5	0.064 (2)	0.050 (2)	0.069 (2)	−0.0058 (15)	0.0380 (17)	−0.0011 (15)
C6	0.0508 (18)	0.0460 (18)	0.0561 (18)	0.0013 (13)	0.0323 (15)	0.0034 (14)
C7	0.0473 (16)	0.0457 (18)	0.0588 (19)	0.0002 (13)	0.0306 (15)	−0.0036 (14)
C8	0.0564 (18)	0.0448 (19)	0.065 (2)	0.0001 (14)	0.0373 (16)	−0.0009 (15)
C9	0.0535 (18)	0.048 (2)	0.063 (2)	−0.0011 (14)	0.0335 (16)	−0.0016 (15)
C10	0.069 (2)	0.058 (2)	0.079 (3)	−0.0047 (17)	0.049 (2)	−0.0035 (18)
F1	0.146 (3)	0.0803 (18)	0.121 (2)	0.0129 (16)	0.105 (2)	−0.0090 (15)
F2	0.125 (2)	0.0773 (18)	0.138 (3)	−0.0305 (16)	0.092 (2)	0.0011 (16)
F3	0.112 (2)	0.0859 (19)	0.0818 (18)	−0.0116 (15)	0.0440 (15)	0.0191 (13)
F4	0.0676 (15)	0.096 (2)	0.139 (3)	−0.0226 (13)	0.0460 (15)	0.0177 (17)
F5	0.169 (3)	0.0515 (16)	0.173 (3)	−0.0089 (16)	0.133 (3)	−0.0034 (16)
O1	0.0897 (19)	0.0399 (14)	0.102 (2)	0.0042 (12)	0.0704 (16)	−0.0031 (12)
O2	0.0793 (17)	0.0555 (16)	0.0924 (19)	0.0030 (12)	0.0633 (15)	−0.0029 (13)

### Geometric parameters (Å, º)

**Table:**

C1—C2	1.379 (5)	C6—C7	1.479 (5)
C1—C6	1.383 (5)	C7—O1	1.290 (4)
C1—H1	0.9300	C7—C8	1.396 (5)
C2—F1	1.344 (5)	C8—C9	1.389 (5)
C2—C3	1.374 (6)	C8—H8A	0.9700
C3—C4	1.367 (6)	C8—H8B	0.9700
C3—H3	0.9300	C9—O2	1.262 (4)
C4—F2	1.353 (5)	C9—C10	1.512 (5)
C4—C5	1.381 (5)	C10—F3	1.316 (5)
C5—C6	1.393 (5)	C10—F5	1.327 (5)
C5—H5	0.9300	C10—F4	1.328 (4)
			
C2—C1—C6	118.8 (3)	O1—C7—C8	120.7 (3)
C2—C1—H1	120.6	O1—C7—C6	115.8 (3)
C6—C1—H1	120.6	C8—C7—C6	123.5 (3)
F1—C2—C3	118.5 (3)	C9—C8—C7	119.2 (3)
F1—C2—C1	118.4 (4)	C9—C8—H8A	107.5
C3—C2—C1	123.1 (4)	C7—C8—H8A	107.5
C4—C3—C2	116.4 (3)	C9—C8—H8B	107.5
C4—C3—H3	121.8	C7—C8—H8B	107.5
C2—C3—H3	121.8	H8A—C8—H8B	107.0
F2—C4—C3	118.5 (3)	O2—C9—C8	125.5 (3)
F2—C4—C5	117.9 (4)	O2—C9—C10	114.0 (3)
C3—C4—C5	123.7 (4)	C8—C9—C10	120.5 (3)
C4—C5—C6	118.0 (4)	F3—C10—F5	106.9 (4)
C4—C5—H5	121.0	F3—C10—F4	104.9 (3)
C6—C5—H5	121.0	F5—C10—F4	107.1 (3)
C1—C6—C5	120.0 (3)	F3—C10—C9	111.8 (3)
C1—C6—C7	121.5 (3)	F5—C10—C9	114.1 (3)
C5—C6—C7	118.4 (3)	F4—C10—C9	111.5 (3)
			
C6—C1—C2—F1	−179.9 (3)	C5—C6—C7—O1	−11.0 (5)
C6—C1—C2—C3	0.8 (6)	C1—C6—C7—C8	−12.7 (5)
F1—C2—C3—C4	−179.9 (4)	C5—C6—C7—C8	168.3 (3)
C1—C2—C3—C4	−0.6 (6)	O1—C7—C8—C9	0.4 (5)
C2—C3—C4—F2	−179.5 (4)	C6—C7—C8—C9	−178.9 (3)
C2—C3—C4—C5	0.4 (6)	C7—C8—C9—O2	−0.3 (6)
F2—C4—C5—C6	179.4 (3)	C7—C8—C9—C10	178.1 (3)
C3—C4—C5—C6	−0.5 (6)	O2—C9—C10—F3	59.0 (4)
C2—C1—C6—C5	−0.9 (5)	C8—C9—C10—F3	−119.5 (4)
C2—C1—C6—C7	−179.9 (3)	O2—C9—C10—F5	−179.5 (3)
C4—C5—C6—C1	0.7 (5)	C8—C9—C10—F5	1.9 (5)
C4—C5—C6—C7	179.8 (3)	O2—C9—C10—F4	−58.1 (4)
C1—C6—C7—O1	168.0 (3)	C8—C9—C10—F4	123.4 (4)

### Hydrogen-bond geometry (Å, º)

**Table:**

*D*—H···*A*	*D*—H	H···*A*	*D*···*A*	*D*—H···*A*
C3—H3···O2^i^	0.93	2.53	3.462 (5)	177

Symmetry code: (i) *x*−1/2, −*y*+1/2, *z*+1/2.
